# Notices

**Published:** 1866-07

**Authors:** 


### NOTICES.

To Southern Subscribers in the good old times of light taxes, cheap living and universal and uninterrupted prosperity we give notice, that mail facilities ceased just after the July number of 1861 was distributed ; we kept the subsequent numbers from August to December, both included, bound in one cover ; they will be sent to each subscriber who will send us their present address.

The contents of the Journal of Health from January 1866 to July inclusive are :

What is Cholera ?
Its very first Symptoms.
What to do.
Signs of Recovery.
Danger of Stimulants.
Danger of Self medications.
Homœopathic Treatment.
Farmer's Houses.
Where to Build.
Miasma and its Laws.
Cellars in Dwellings.
Smoky Chimneys.
Water conveniences.
Water Closets.
Ice Houses.
Stables.
Kitchens.
Chambers.
Shade Trees.
Barns.
Water Pipes.
Crazy Farmers, Why.
Wives Overworked.
Daughters ill Health.
Surprise Parties.
Shams.
Potatoes as Food.
To stop Coughing.
Foul Odors.
Preaching Easily.
Domestic Cleanliness.
Ventilation.
Laws of Cholera.
Quarantine.
Cholera Prevented.
Fear of Cholera.
Emergencies.
Extemporaneous Surgery.
Curiosities of Breathing.
Symptoms.
Cellars.
Filth and Purity.
Weights and Measures.
Medical Terms.
Biliousness.
Trichiniasa.
Night Work.
Night and Disease.

All new subscribers must begin with the January number. The January and February numbers are taken up with the subject of the Cholera, and will be sent post paid for 30 cents ; the object of the article is to teach the reader to know what are always the first far off symptoms of Cholera ; when he will be in reality cured, without any medicine whatever if these symptoms are *first* attended to ; what are the more advanced symptoms ; and what is considered the most infallible remedy at this state, applicable to all cases, as a means of arresting the disease until a physlcian can be called ; the folly of *taking* anything as a preventive of Cholera ; the reason why a so-called preventive will certainly increase the chances of an attack; the certain sign of commencing recovery ; the absolute importance of securing the services of a physician in all cases, where attention was not given to the first symptoms ; how easy it is to know these first symptoms ; the importance of remembering that, as the Cholera, if it comes this year, may assume a different phase, from that of former times, it is not safe to rely on any old remedy, nor to rely on any one's advice but that of a practising physician, and that no confidence whatever ought to be placed on any newspaper receipt, because what might be appropriate in former years, and other places, cannot be relied on for this year in this locality, as the disease is known to present different different types in different places and different seasons.

Persons who fail to receive their numbers must apply for them during the month for which it is published, otherwise send twelve cents for a duplicate; remembering, however, that it is always supplied as a courtesy, and not as a right, for the Publisher's responsibility ends with depositing the Journal in the New York Post Office. It is curious to observe that nine persons out of ten who do not receive their number take it for granted that the reason is the neglect of the Publisher in not mailing it. In mailing the Journal, all the numbers of each State are sent in one package to the distributing post office of that State, and that a failure to receive a number is owing to the Postmaster at the distributing office, or to the Postmaster at the office where the Journal is received, and it is next to impossible to be the fault of the New York Post Office, for if one copy of the Journal is received in any one State, all the copies must have been sent to that State; and if one copy is received at any one office where four or more are due, all the copies must have gone there, as all were sent to that post office in one package. So when your copy is missing, go to your own Postmaster before writing to the Publisher, "If I can't receive my paper regular, I don't want it sent any more." Whenever a letter comes indicating a little temper the Publisher lays it aside with a smile, saying, "O! he's young; don't know much." Persons sending subscriptions or for missing numbers must address simply,

"Hall's Journal of Health,
New York."

and the publisher will get it; those wanting medical advice must address the editor thus:

"Dr. W. W. Hall, New York."

The Editor's office is at No. 2 West 43d St., New York, where he may be quite surely found any day until 2 P. M.; no one need come a minute after nine o'clock at night, because he is then in bed; and often he gets sleepy as soon it is dark, and it is never safe to take advice of a sleepy Doctor; and even when we are wide awake how little do we know! The biggest fool on this planet is the man who thinks he knows much of anything except himself, and that is a knowledge which most people are not particularly anxious to communicate; but such knowledge as we have we are willing to impart for a liberal consideration, for unless we charge pretty well for the small stock on hand, we would soon run out of provender. We wish everybody had as good an opinion of us as a correspondent, writing June 1st., 1866: "You have done for me more than all the physicians I ever saw put together." And after getting well he wants to show himself, most, people would better prefer showing themselves first.

WANTED TO GIVE To whomsoever will procure thirty-six paying subscribers to Hall's Journal of Health for 1866, a Wheeler and Wilson Sewing Machine, costing cash $56. This Machine will sew all kinds of fabrics, and is the cheapest and best manufactured of its kind. Specimen numbers will be sent post paid for 10 Cents.

SLEEP.—A correspondent two thousand miles away, writes thus enthusiastically about our book on "sleep" sent pp. for $1.60. "Allow me to express my everlasting obligations to you for having saved me from the dark gulf of perdition and despair, through the means of your book entitled SLEEP. I followed your common sense rules and directions, and to-day can hold up my head like a man."

PORK WORMS.—The eminent medical professors who were sent to Germany to investigate the subject, state that Trichiniasis is everywhere either extinct or dying out; that the epidemic at Herdensleben was the result of a remarkable concourse of unfortunate circumstances, and that a heat of one hundred and sixty-six degrees is fatal to the worm.

THE LITTLE CORPORAL, says the Chicago Journal, has a circulation of thirty thousand copies, and is the best child's paper, at one dollar a year.

THE NATION, published twice a week, five dollars a year, by. J. H. Richards, 130 Nassau St. Each number contains topics of the day, proceedings of Congress, literary notes and notices, scientific articles, with much in addition of other matter. No. 48. for example, has "The Usury Laws, Paris Gossip, Moral of Memphis Riots, Our System of Legislation, Popular Influence of Moral Censorship, Influence of Towns over Counties at Home and Abroad; every number contains a large amount of well written articles on practical and suggestive subjects.

MERRY'S MUSEUM FOR JUNE, 172 William St., New York—Contains: Silverstone and Slate, by Kruna; Harry and his Dog, by Pearl Peveril; *Wild Oats*, by Sophie May; Uncle Godfrey's Lectures; Short Sermons to News Boys, by Rev. Charles L. Brace; Catching Rats *vs.* Study, by Uncle Tim; A Story whose end is in a Picture Gallery, by the author of Philip Snow's War; Who made the Flowers? Merry's Monthly Chat and Fleta Forrester's Puzzle Drawer.

TEMPERANCE.—The New York and Brooklyn Temperance Alliance is to promote entire abstinence from all intoxicating drinks, by lectures, visitations, tracts, and special efforts to save the young, by the formation of juvenile Temperance Societies in Sabbath and day schools. President Hon. S. Booth, Mayor of Brooklyn, Miss Thos. Davis and George Thompson are employed as Temperance Missionaries, and are indefatigable in their labor of love, extending to the generous sailor their personal efforts in distributing tracts, holding meetings and talking personally with our Jack Tars. The Society is in need of funds, which can be sent to William E. Dodge, Esq.,—the liberal friend of all good measures—or T. B. Wells, Treasurer, 389 Broadway, New York. Religious newspapers for distribution on board ships, for sailors to read on their long voyages will be thankfully received at the same place, or will be called for at the residence of our citizens who will send their address.

